# Investigating the immunomodulatory nature of zinc oxide nanoparticles at sub-cytotoxic levels *in vitro* and after intranasal instillation *in vivo*

**DOI:** 10.1186/s12951-015-0067-7

**Published:** 2015-02-03

**Authors:** Shruti R Saptarshi, Bryce N Feltis, Paul FA Wright, Andreas L Lopata

**Affiliations:** Molecular Immunology Group, College of Public Health, Medical and Veterinary Sciences, Centre for Biodiscovery and Molecular Development of Therapeutics, James Cook University, Building 21, Molecular Sciences, James Cook Drive, Douglas Campus, Townsville, QLD 4811 Australia; School of Medical Sciences, RMIT University, Melbourne, VIC Australia; Department of Materials Engineering, Monash University, Melbourne, VIC Australia

**Keywords:** Heme oxygenase-1, Reactive oxygen species, IL-8, Intranasal instillation, A549 cells

## Abstract

**Background:**

This study evaluates the time-dependent pro-inflammatory response of the model human lung epithelial cells (A549) to industrially relevant zinc oxide nanoparticles (ZnO NPs). In terms of toxicity, ZnO-NPs are categorised into the group of high toxicity nanomaterials. However information on pro-inflammatory potential of these NPs at sub-toxic concentrations is limited. Understanding how cellular defense mechanisms function in the presence of sub-cytotoxic concentrations of these NPs is vital. Moreover, there is an urgent need for additional *in vivo* studies addressing pulmonary toxicity due to accidental inhalation of ZnO NPs.

**Results:**

Exposure to sub-cytotoxic ZnO NP concentrations (20 μg/mL) induced significant up-regulation of mRNA for the pro-inflammatory cytokine IL-8 and redox stress marker heme oxygenase-1, along with increased release of IL-8. The highest pro-inflammatory response was recorded after 4 to 6 hr exposure to ZnO NPs over a 24 hr period. Pre-treatment of A549 cells with the sulfhydryl antioxidant N-acetyl cysteine (at 5 mM) resulted in significant reduction of the up-regulation of inflammatory markers, confirming the role of reactive oxygen species in the observed immunomodulatory effects, independent of cytotoxicity. Furthermore, we report for the first time that, intranasal instillation of a single dose (5 mg/kg) of pristine or surfactant-dispersed ZnO NPs can cause pulmonary inflammation, already after 24 hr in a murine model. This was confirmed by up-regulation of eotaxin mRNA in the lung tissue and release of pro-inflammatory cytokines in the sera of mice exposed to ZnO NPs.

**Conclusion:**

Our study highlights that even at sub-cytotoxic doses ZnO NPs can stimulate a strong inflammatory and antioxidant response in A549 cells. ZnO NP mediated cytotoxicity may be the outcome of failure of cellular redox machinery to contain excessive ROS formation. Moreover exposure to a single but relatively high dose of ZnO NPs via intranasal instillation may provoke acute pulmonary inflammatory reactions *in vivo.*

**Electronic supplementary material:**

The online version of this article (doi:10.1186/s12951-015-0067-7) contains supplementary material, which is available to authorized users.

## Background

Engineered zinc oxide nanoparticles (ZnO) NPs offer versatility and unique physicochemical properties that have a vast array of commercial applications, and are widely used in cosmetics and sunscreens, because of their excellent UV filtering properties and aesthetic appeal [1]. Interestingly, despite high production volumes and a broad application base, there is always a possibility of accidental exposure to NPs. This can result in unintentional side-effects caused due to the propensity of these materials to demonstrate biological reactivity [2]. One particular concern is exposure to high concentrations of ZnO NPs via inhalation in the occupational setting, where nanomaterials are manufactured or added to products [3]. Improved understanding of how cells interact to the presence of NPs in their micro-environment can help ascertain either the toxic potential or usefulness of these materials for future biomedical applications.

ZnO NP mediated cytotoxicity has been previously reported in several *in vitro* test systems including immune cells, lung epithelial cells, colorectal epithelial adenocarcinoma (CaCo-2) cells etc. [4-8]. Other studies have shown that ZnO NPs can cause cytotoxicity via apoptosis [9-12] and also genotoxicity [13-15]. Despite some contradictory reports, the main paradigm explaining ZnO NP cytotoxicity appears to be their tendency to dissolve, resulting in the generation of Zn^2+^ ions, with an associated generation of reactive oxygen species (ROS) [8,16]. Extracellular dissolution of ZnO NPs and release of Zn^2+^ ions has been reported to induce cell death in human T-cell leukaemia (Jurkat) cells [17], mouse macrophage (Ana-1) cells [18] or apically-exposed rat alveolar epithelial cell monolayers [19]. Recently, it was proposed that ZnO cytotoxicity requires direct particle-cell contact and uptake, resulting in the release of high intracellular concentrations of Zn^2+^ ions from ZnO dissolution within lysosomes and late endosomes [20-22]. This hypothesis has also been suggested for the *in vivo* scenario where intratracheally instilled ZnO NPs were shown to induce eosinophilia, and pulmonary fibrosis in exposed rodents [23] which may be mediated via ROS formation [24-26]. ZnO NPs inducing an overproduction of ROS can also result in apoptosis in rat retinal ganglion cells, [12] but in case of ZnO NP-exposed Ana-1 cells, contrasting results were observed [18]. Apart from cytotoxicity, inflammatory potential of ZnO NPs has also been investigated [3,27]. Zinc is an essential trace element and plays an important role in regulating cellular metabolism [28,29]. Therefore, it is also possible that increased exposure to ZnO NPs may cause immunomodulation. Currently, there is a lack of a clear understanding of how the inflammatory potential of ZnO NPs, their dissolution characteristics and subsequent ROS generating capacity are interlinked together particularly at the sub-cytotoxic level. Moreover, the bio-reactivity of NPs may be greatly influenced by their biological environment [2].

Systemic deposition of NPs *in vivo* may lead to their interaction with immune cells resulting in immunomodulatory consequences. Intratracheal instillation of ZnO NP has been reported to cause classic pulmonary oxidative-inflammatory responses [23,30-32]. Inhalation of ZnO NPs may lead to their translocation to the central nervous system and may also interfere with zinc homeostasis leading to pulmonary toxicity [33,34]. Intratracheal instillation of NPs is not a route of exposure in the occupational setting and can cause less homogenous and focally distributed deposition of the nanomaterial in the lungs. Conversely, intranasal instillation can be used to deliver NPs to the upper as well as lower respiratory tracts. Although intranasal instillation of ZnO NPs has been shown to likely cause injury to the olfactory epithelium of exposed rats, [35] probable pulmonary side-effects and systemic inflammatory responses resulting from this route of exposure have not been investigated before. Furthermore, additional *in vivo* studies are required to determine “no-observed-adverse-effect-levels” of accidental exposure to NPs [36].

In the present study, we have assessed the immunomodulatory potential of industrially manufactured ZnO NPs, focusing on three different particle sizes (30, 80 and 200 nm) and dispersal states using a commercial surfactant to investigate the effect of aggregation. We have made use of human lung epithelial (A549) cells for the *in vitro* exposure study as they are representative of the alveolar type II cells in lungs and are widely used in nanotoxicology. In addition, based on the findings from our *in vitro* study, we chose two ZnO NPs (pristine 30 nm and surfactant-dispersed 30 nm) for an acute high dosage intranasal exposure study in mice. The aims of the study were 1) to systematically investigate the time-course responses over 24 hr of A549 cells exposed to sub-cytotoxic levels of ZnO NPs, to further understand the kinetics and elucidate the mechanisms underlying their observed immunomodulatory and cytotoxic effects, and 2) evaluate the effects of an acute exposure of a single high dose of ZnO NPs via the intranasal route of airway exposure *in vivo*.

## Results

### Cytotoxicity profile

**Figure 1:**
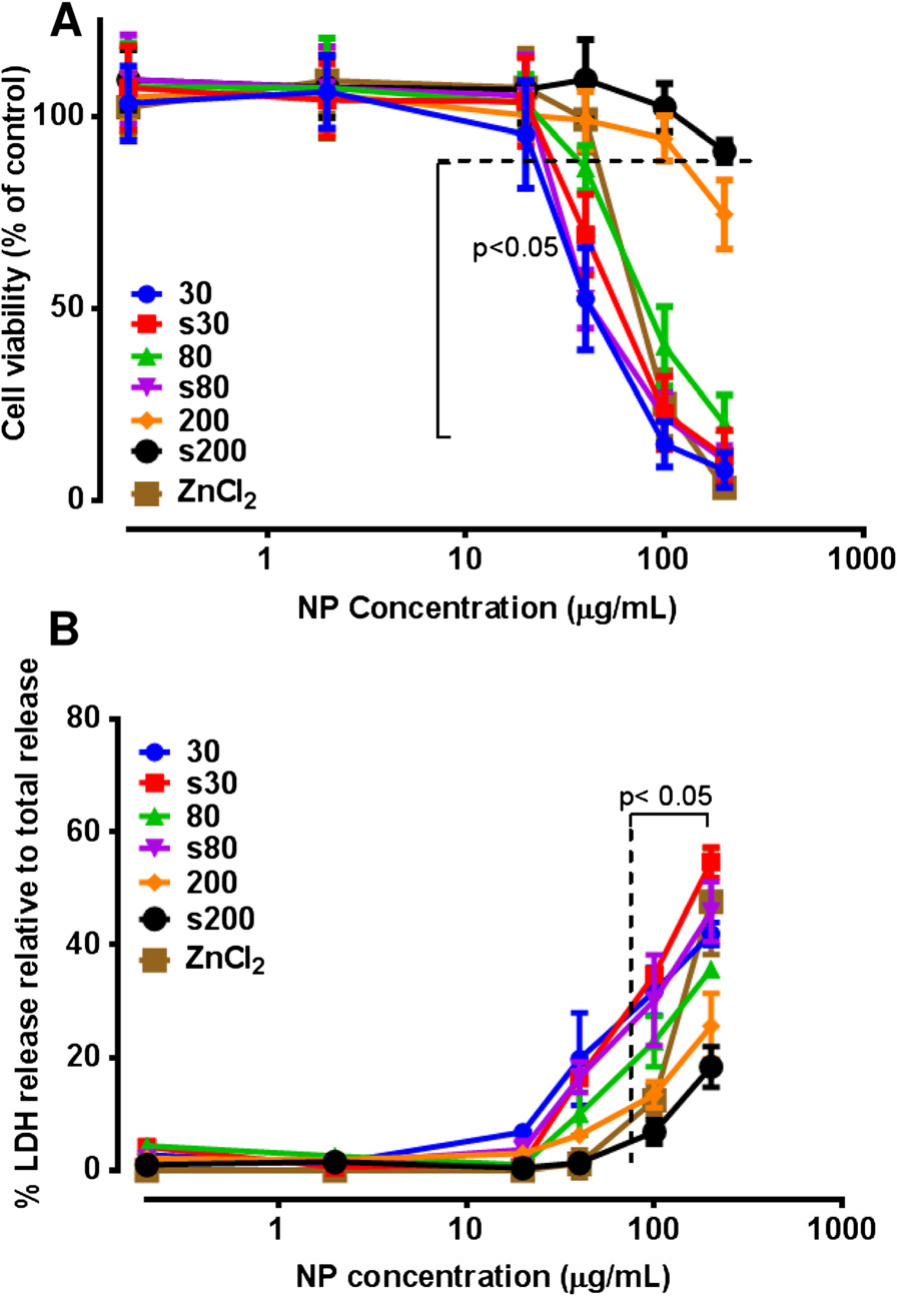
**Cytotoxicity profile of pristine and surfactant-dispersed ZnO NPs after 24 hr exposure of human lung epithelial A549 cells, using the (A) MTS assay and (B) lactate dehydrogenase release assays.** Cytotoxicity was dependent on NP size and dispersal state. Concentrations at or below the dotted line (MTS assay) and beyond the dotted line (LDH release assay) were significantly different from untreated control cells. Values are expressed as mean ± SEM (n = 5 separate experiments).

Cytotoxicity observed for all ZnO NPs was concentration dependent and similar to ZnCl_2_. Surfactant-dispersed 80 nm ZnO NPs demonstrated greater cytotoxicity than their pristine counterpart when comparing the effective concentrations that produced 50% cell death (EC50) (Figure 1A). Whereas, the cytotoxicity observed for the pristine particulates was marginally greater (p < 0.05) than the surfactant-dispersed 200 nm ZnO at the highest tested concentration. Overall, the surfactant-dispersed 200 nm ZnO particulates were found to be the least cytotoxic material, achieving less than an EC25 response at the highest tested dose (200 μg/mL). This surfactant by itself has previously been shown to be non-cytotoxic over this same dose-range [6]. LDH release was measured after 24 hr exposure and the results were concentration dependent (Figure 1B), but less sensitive than MTS, as all NPs up to the maximum dose of 200 μg/mL showed a higher degree of similarity in LDH release. LDH values were significantly different to untreated control cells at 100 μg/mL, compared to the MTS assay achieving statistical significance at 30 μg/mL.

### Up-regulation of pro-inflammatory IL-8 and stress-responsive HO-1 genes

**Figure 2:**
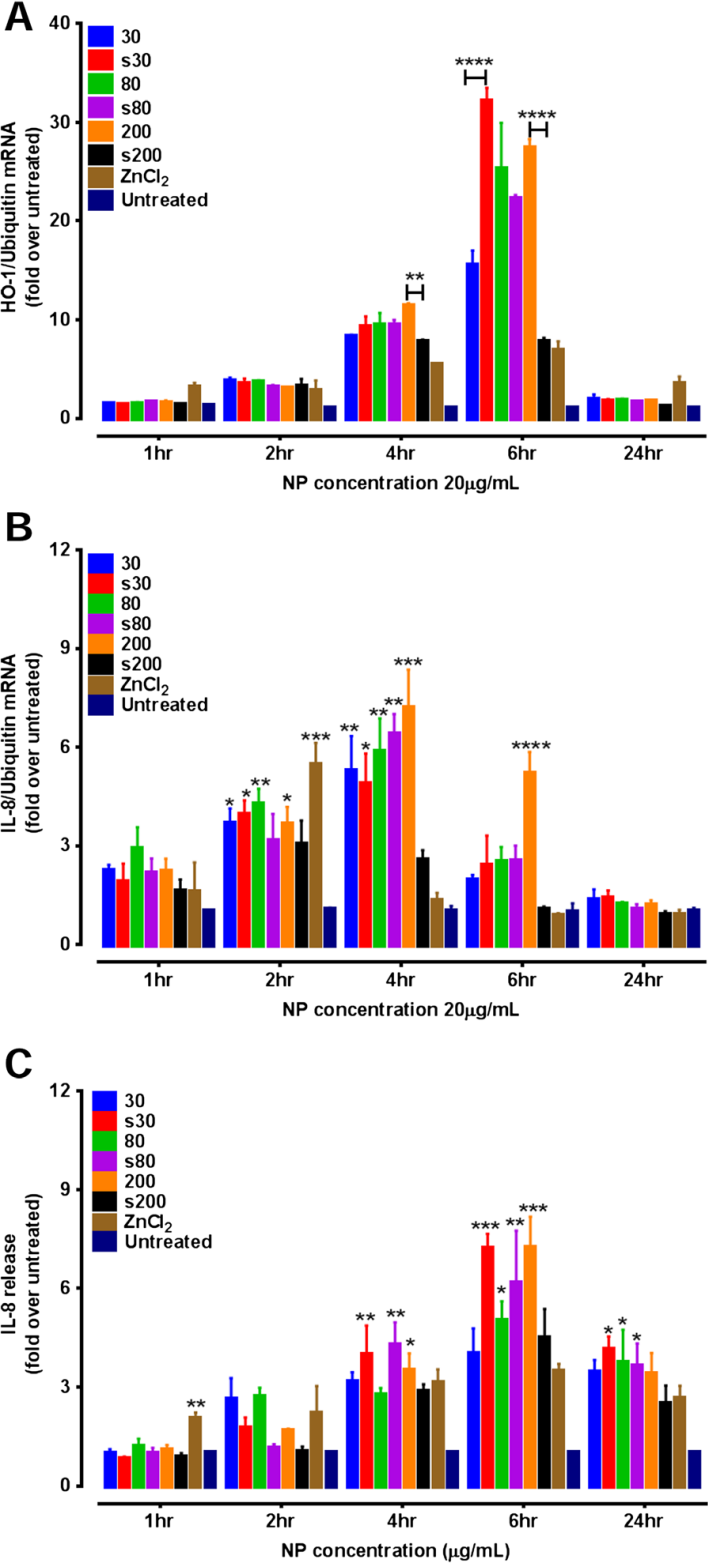
**ZnO NP mediated pro-inflammation in A549 cells exposed to the sub-cytotoxic dose (20 μg/mL) over 24 hr.** **(A)** ZnO NP induced up-regulation of HO-1 mRNA indicative of a strong antioxidant response in A549 cells. Highest HO-1 expression was seen at 6hr which was a 30-fold increase in gene expression compared to untreated cells at 6hr (p<0.001). Block bars compare the statistical significance between pristine or surfactant-dispersed solutions for each ZnO NP size. **(B)** mRNA over expression of the pro-inflammatory cytokine IL-8 in A549 cells stimulated with 20 μg/mL of ZnO NPs at 1, 2, 4, 6 or 24hr. Maximum expression of IL-8 gene was recorded at 4hr. **(C)** Release of pro-inflammatory cytokine IL-8 by ZnO NP-exposed A549 cells at 1, 2, 4, 6 or 24 hr. Statistical significance is shown in **(B)** and **(C)** compared to untreated cells at each time point. *p<0.05,**<0.01,***p<0.001, ****p<0.0001. Data are expressed as mean ±SEM of the fold levels compared to untreated controls at each time point (n=3) separate experiments, each performed with triplicates).

Systematic investigation of HO-1 up-regulation as a function of time revealed that, the activation started at 4 hr after exposure, peaked at 6 hr and had subsided by 24 hr, when compared to basal levels of HO-1 gene activation in untreated cells (Figure 2A). At 4 hr, HO-1 gene up-regulation was similar for all zinc exposures and ranged from 6 to 12-fold. Overall, the highest up-regulation at the 6 hr time point was induced by s30 ZnO NPs, showing a 30-fold increase compared to untreated cells. In decreasing order, HO-1 gene up-regulation at 6 hr was caused by: s30 > 80 ~ s80 ~ 200 > 30 > s200 ~ ZnCl_2_.

In the case of IL-8 gene expression, maximum stimulation occurred at 4 hr exposure when compared to the untreated control cells. IL-8 induction from all particulates, except surfactant-treated 200 nm ZnO, ranged from 5 to 7-fold that of the untreated control (Figure 2B). However, after 24 hr exposure IL-8 gene expression had returned to basal levels. Interestingly, ZnCl_2_ treated cells, after 2 hr exposure, showed IL-8 gene up-regulation that was significantly higher (p < 0.05) than all treatments except s80 and s200 ZnO NPs. At all other time points ZnCl_2_ induced IL-8 gene mRNA levels were similar to those of untreated control cells. Pristine ZnO 200 nm particles were the most potent at both 4 and 6 hr. Surfactant-dispersed 200 nm particulates did not cause significant IL-8 gene up-regulation. Moreover, replacement of the ZnO NP containing cell culture medium with fresh medium 1 hr after exposure to ZnO NPs did not stimulate expression of HO-1 or IL-8 gene (data not shown). This could be because, 1 hr is insufficient time for significant cellular uptake of ZnO NPs, and we have previously demonstrated the low solubility of these NPs in cell culture medium. Clearly, the effects on gene expression require cell uptake and intracellular dissolution.

### IL-8 release

A gradual increase in IL-8 levels in culture medium was already apparent after 4 hr exposures for all NPs, except pristine ZnO 80 and surfactant-dispersed 200. The largest IL-8 release was seen at 6 hr, where all particulates showed at least 4 fold increases in IL-8 release, as compared to the untreated control cells. (Figure 2C). IL-8 levels were not significantly altered at the earlier 1 and 2 hr time points for the ZnO NPs and particulates except zinc.

### Effect of sulfhydryl antioxidant

**Figure 3:**
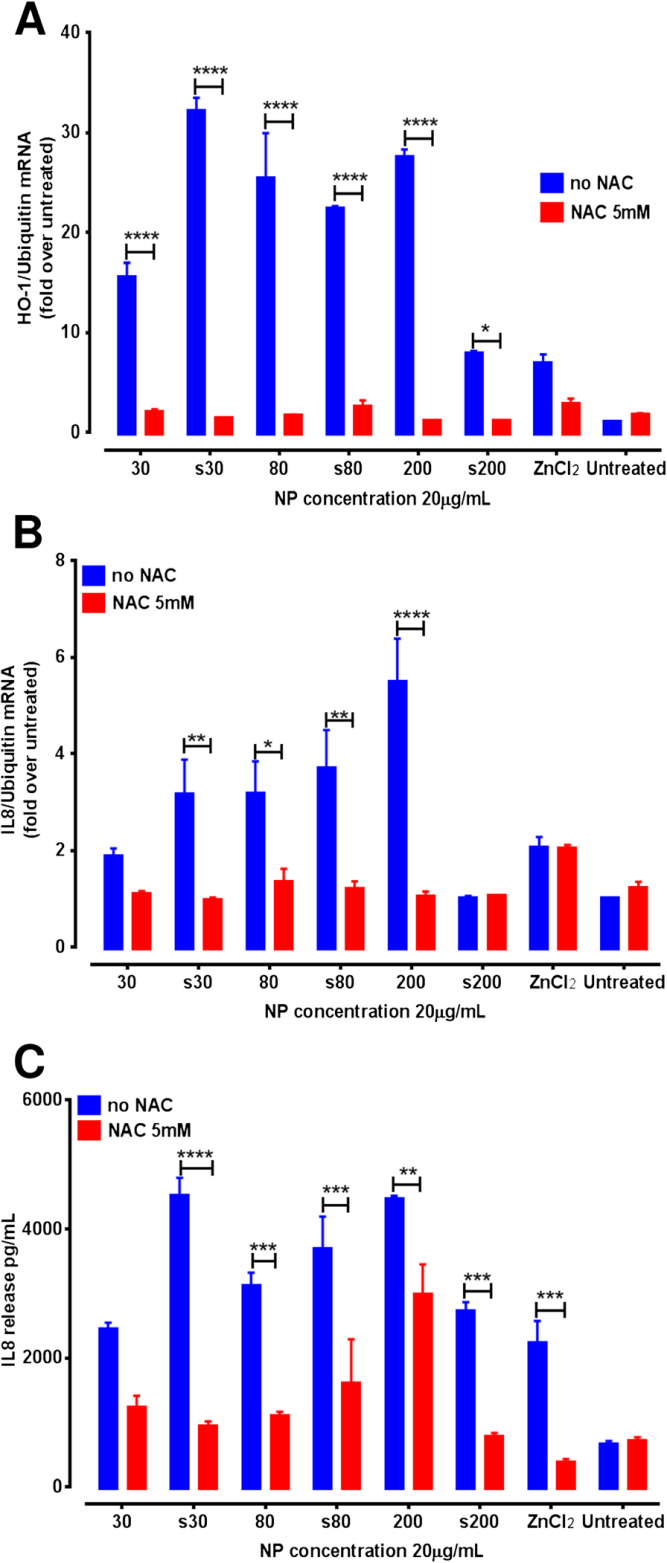
**Mitigation of the up-regulation of (A) HO-1 mRNA expression and (B) IL-8 mRNA expression (C) IL-8 cytokine release in antioxidant treated A549 cells after exposure to ZnO NPs (20 μg/mL) for 6 hr.** Statistical significance after comparing no NAC or 5 mM NAC cells where *p < 0.05, ** < 0.01, ***p < 0.001, ****p < 0.0001. Data are expressed as mean ± SEM of the fold levels compared to untreated controls at each time point (n = 3) separate experiments, each performed with triplicates).

The highest mRNA expression for HO-1 and IL-8 genes was seen at 6 hr and 4 hr post-exposure, respectively. To confirm the role of ROS in the observed immunomodulatory responses, A549 cells were pre-treated for 1 hr with 5 mM of NAC followed by exposure to ZnO NPs for 6 hr (when IL-8 gene expression was also still marginally up-regulated and IL-8 release was progressively increasing). The concentration of NAC used for this experiment did not induce cytotoxicity (108 ± 1.5% of control). Pre-treatment with NAC dramatically reduced the ZnO-induced up-regulation of mRNA expression levels for both HO-1 and IL-8 genes (Figure 3A & B). IL-8 gene expression increases were not observed for s200 ZnO or ZnCl_2_-treated cells. Levels of IL-8 release by NAC pre-treated A549 cells following exposure to ZnO and ZnCl_2_ were also found to be lower than exposed cells without NAC pre-treatment (Figure 3C).

### Cell signalling

**Figure 4:**
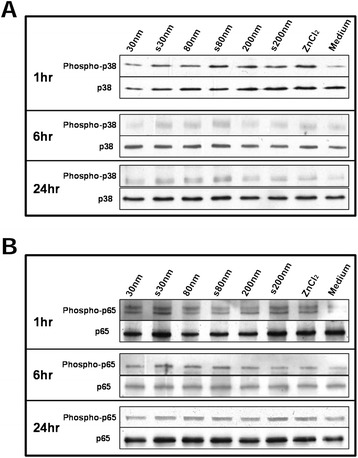
**Immunoblotting showing (A) phosphorylation of p38 protein, belonging to the redox-sensitive p38 Mitogen activated protein kinase (MAPK) pathway and (B) p65 protein, involved in NFκB activation, after exposing A549 cells to 20 μg/mL dose of ZnO NPs.**

ZnO NPs were able to induce a rapid phosphorylation of the p38 protein, a representative of the MAPK signalling pathway cascade (Figure 4A). Exposure of A549 cells to 20 μg/mL of ZnO particulates or ZnCl_2_ also induced phosphorylation of the p65 protein, which is pivotal to NFκB signalling (Figure 4B). Characteristic bands of phosphorylated p65 protein were observed after 1 hr exposure to ZnO NPs or ZnCl_2_ and were significantly stronger than those observed at 6 or 24 hr. Cells exposed to medium-only served as a negative control.

### *In vivo* short-term exposure to high dose ZnO-NP

**Figure 5:**
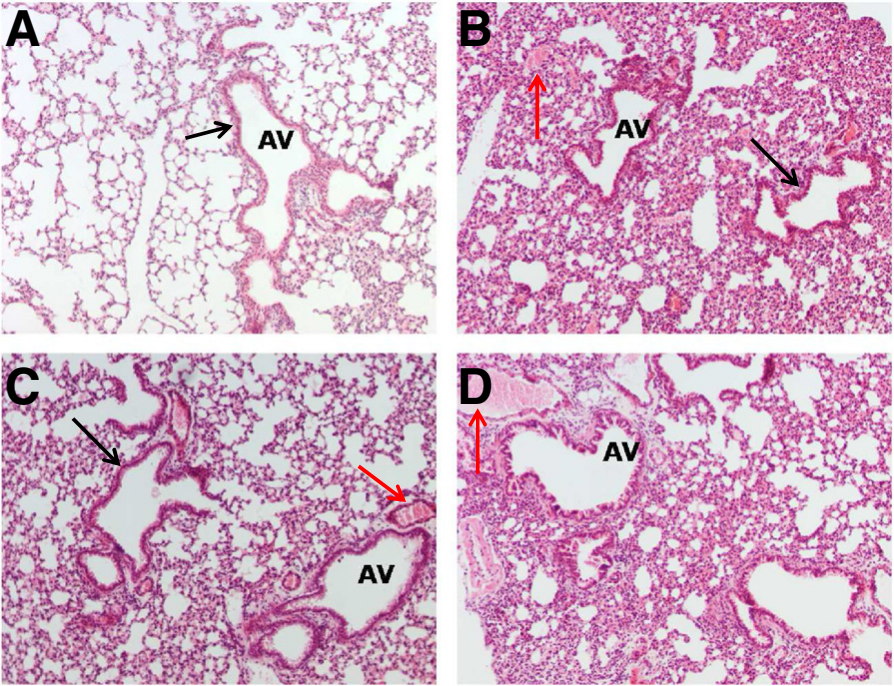
**H&E staining of lung sections of mice treated with (A) vehicle control, (B) LPS, (C) pristine 30 nm ZnO NPs and (D) surfactant-dispersed 30 nm ZnO NPs via intratracheal instillation.** AV indicates the alveolar region of the lung. Red arrows indicate haemorrhage and black arrows demonstrate thickened bronchial walls.

**Figure 6:**
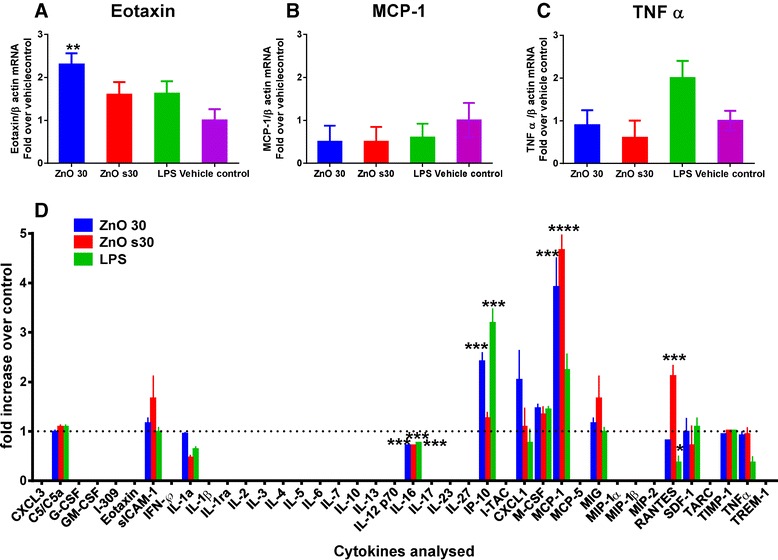
**qPCR analysis of mRNA levels for inflammatory markers (A) eotaxin, (B) MCP-1 and (C) TNFα in the lung tissues of treated mice. (D)** Proteome profile array of pro-inflammatory cytokines quantified in pooled sera from each experimental group. Statistical significance is compared to vehicle control. Pink bars for A,B,C and dotted line for D represent the vehicle control. Data are expressed as mean ± SEM (n = 12) from two separate experiments.

Figure 5 demonstrates haematoxylin and eosin staining of the lung sections of the control group (Figure 5A) treated with water showing normal morphology. In contrast, lung sections of ZnO NP exposed mice (Figure 5C & D) show severe inflammatory infiltration in the alveoli and peri bronchial regions. The bronchial and vascular walls also appear to be thickened (black arrows). A moderate level of internal haemorrhage was also observed 24 hr after NP challenge when comparing lung tissue section of control mice (red arrows). RNA extracted from the lung tissue of ZnO NP treated mice was used for qPCR analysis of key pro-inflammatory markers. A significant up-regulation of the eotaxin gene was observed in mice treated with pristine 30 nm ZnO NPs compared to mice exposed to vehicle control (Figure 6A). In contrast, at the end of 24 hr ZnO NPs did not cause significant up-regulation of pro-inflammatory TNFα and MCP-1 genes in the lung tissue of treated mice (Figure 6B & C). Cytokine profiling of pooled mouse serum from each experimental group revealed the presence of pro-inflammatory chemokines (Figure 6D). Significant levels of MCP-1 protein were detected in the pooled sera of mice treated with either ZnO NP solutions. Pristine 30 nm ZnO NP treated mice showed significantly elevated levels of the chemokine IP-10, while 30 nm sZnO NPs caused an increase of CCL5 (RANTES), known to recruit leukocytes to inflammatory sites. Interestingly, eotaxin was not detected in the sera of ZnO NP exposed animals. Overall, the *in vivo* inflammatory responses observed for NP treatment was distinct from that observed for LPS treatment.

## Discussion

Detailed knowledge of the ability of cells to deal with both cytotoxic and sub-cytotoxic doses of ZnO NPs can help in understanding mechanisms of cellular responses to a range of concentrations of these NPs to which workers may be exposed. The present study highlights effect of physical parameters, such as dispersal state and agglomerate size of ZnO NPs, along with duration of contact with cells, on NP immunomodulatory and cytotoxic potential.

The cytotoxicity of ZnO NPs was quantified using two different assays, the MTS assay which measures the mitochondrial metabolism of metabolically-viable cells, and the LDH release assay which quantifies the release of this cytosolic enzyme from non-viable cells after cell lysis. ZnO NPs cytotoxicity was concentration, size and time dependent. These results are in agreement with cytotoxic responses to identical NPs of human monocytes and macrophages (THP-1 cells), as demonstrated in previous studies by our group [6]. Surfactant-dispersed and undispersed 200 nm particulates used in our study displayed lower cytotoxicity at the highest tested concentrations although the surfactant compound by itself has been previously shown to be non-toxic over the same concentration rage *in vitro* [6]. In the 80 nm size range, the surfactant-dispersed NPs were more cytotoxic than their undispersed counterparts. Interestingly, for 30 nm NPs, the MTS assay indicated that the pristine 30 nm particles were most cytotoxic, whereas the LDH assay indicated the surfactant-dispersed material was more cytotoxic. It should be noted that the surfactant may alter the membrane permeability of the cells to some extent which could be responsible for the observed inconsistencies in the cytotoxicity profiles of these two assays. Additionally, the LDH assay was also less sensitive than the MTS assay, as the maximum LDH release for the highest concentration of ZnO NPs used at 24 hr was found to be only 50% of LDH release from total cell lysis. Relating this to other measures of cell death, DAPI staining of A549 cells exposed to a cytotoxic dose of ZnO NPs for 24 hr (data not shown) revealed characteristic condensation of the nuclear material, as is typically observed during apoptosis, which has also been previously observed in THP-1 monocytes [6].

We next analysed how sub-cytotoxic doses of different sizes and dispersal states of ZnO NPs affected A549 cells over 1, 2, 4, 6 and 24 hr. At these low concentrations, the data should not be confounded by factors expressed during cell death and these should not contribute to the expression of biomarkers used to assess the pro-inflammatory potential of ZnO NPs in the present study. Also, contamination of NP solutions with lipopolysaccharide (LPS) can occur and it is therefore important to distinguish nanoparticle toxicity from endotoxin related effects. We have previously demonstrated that the cytotoxic and inflammatory responses of the same ZnO NPs or LPS were significantly different in an *in vitro* system consisting of THP-1 monocytes [6]. Furthermore, A549 cells used in the present study are known to be relatively insensitive to LPS. This further confirmed that the altered immune responses of ZnO NP exposed A549 cells was not due to LPS-contamination.

ROS generation has been extensively implicated as an important driver of nanotoxicity [37]. However, whether cytotoxicity and ROS formation are interdependent or causally linked needs further investigation. A three tiered oxidative stress response has been previously described for NP-mediated toxicity [16,38]. The lowest level of oxidative stress is characterised by the induction of antioxidant defence genes. This is followed by the second tier where a pro-inflammatory response is initiated. HO-1 and IL-8 genes are representatives of this oxidative stress/pro-inflammatory paradigm. The oxidative stress-responsive HO-1 is primarily involved in the conversion of heme to biliverdin and has been also reported to have anti-inflammatory properties [39]. In our study, we observed a gradual increase in expression levels of HO-1 gene from 2 hr, reaching maximum activation at 6 hr and returning to basal levels at 24 h. These results are in accordance with studies showing similar results, with human endothelial cells exposed to SiO_2_ NPs [40], and A549 cells exposed to Fe_3_O_4_ magnetic NPs [41]. In our study, surfactant-treated 30 nm ZnO NPs maximally induced HO-1 expression, compared to the other NPs studied. This may be because of the enhanced accessibility of these NPs to the cell surface due to the small size and improved dispersal, which may have resulted in greater uptake and subsequent intracellular release of zinc ions. This rapid uptake and release would likely create an imbalance in the cellular antioxidant capacity [42]. It is also worth reiterating that the surfactant may alter membrane permeability to NPs to some extent, which would also potentially increase uptake. The HO-1 and IL-8 results indicate a strong antioxidant response of exposed cells against the ROS generated due to their interaction with ZnO NPs.

Airway epithelial cells offer an important physiological barrier against inhaled matter, and are responsible for releasing a number of crucial cytokines and chemokines. The α chemokine IL-8 is a potent chemo attractant for neutrophils and eosinophils, and a possible protagonist in the progression of airway inflammation [43]. Sub-cytotoxic doses (2 – 8 μg/mL) of ZnO NPs have previously been shown to increase IL-8 mRNA expression and IL-8 release in exposed BEAS-2B cells [3]. Pro-inflammatory effects resulting from exposure to a sub-cytotoxic dose of ZnO NPs have included the release of IL-8 by nasal mucosal cells into the culture medium [14]. In the present study, all tested ZnO NPs were capable of inducing significant up-regulation of IL-8 mRNA in A549 cells, which was maximal after 4 hr exposure compared to untreated cells. Interestingly, the 200 nm ZnO particulates demonstrated a similar response to the s30 ZnO NPs. This may be partially because of the greater heterogeneity in the 200 nm ZnO NPs, which also contains a proportion of both smaller and larger sized particles. Moreover, while surfactant dispersal allows the NPs to remain in solution for longer intervals, pristine NPs tend to agglomerate and sediment rapidly, resulting in enhanced cellular dosing, which may cause increased activation of cellular mRNA, as has been previously reported [44]. Similar observations have been reported for 100 or 20 nm ZnO NP treated HaCaT cells [45]. In conjunction with IL-8 mRNA up-regulation, we also quantified the relative levels of IL-8 protein release into cell culture medium. At low NP doses, A549 cells in our study demonstrated increased expression of IL-8 involving transcriptional activation of NFκB, followed by further stabilisation of IL-8 mRNA by p38 mitogen activated protein kinase pathway [43]. Transcriptional and post-transcriptional regulation of ZnO NP-mediated over expression of the IL-8 gene has previously been reported for BEAS-2B cells, which required phosphorylation of protein p65 [3,46]. Similarly, the p38 MAPK pathway is known to be activated by a number of stimuli including oxidative stress, which can lead to phosphorylation of the p38 protein. In this study, ZnO NPs and ZnCl_2_ treatment induced rapid phosphorylation of p65 (Ser536) and p38 in treated A549 cells after 1 hr exposure, compared to untreated cells, and this returned to basal levels at 6 or 24 hr. Uptake of ZnO NPs may occur via endocytosis [46]. Once internalised into the acidic lysosomes, ZnO NPs dissolution occurs, which can disrupt intracellular zinc homeostasis [21,22]. We have previously demonstrated low rates of dissolution in cell culture medium, both with and without protein supplementation [6,47] (Additional file 1: Table S1). It is therefore unlikely that extracellular dissolution is the driving cause of the pro-inflammatory signals observed for these NPs. Exposure of A549 cells to ZnO NPs for 1 hr followed by washing and supplementing cells with fresh medium and then a further incubation for 4 hr, did not stimulate mRNA over expression of the pro-inflammatory cytokines. Taken together with the dissolution data, indicates that direct continued contact of ZnO NPs with the cells is essential. Furthermore, pre-treatment of A549 cells with NAC not only was able to significantly reduce up-regulation of both the HO-1 and IL-8 genes, but also controlled the release of IL-8 protein. This confirmed participation of ROS in the development of the observed tier 1 and 2 redox responses in A549 cells exposed to a sub-cytotoxic concentration of ZnO NPs.

The lung environment represents a complex biological system. Inhalation or intratracheal instillation of ZnO NPs in rodents can induce increased polymorphonuclear cell counts, eosinophilia and oxidative stress like symptoms [30,31,48]. Our analysis after intranasal instillation of ZnO NPs showed results that were similar to the cytotoxic patterns observed for A549 cells. ZnO NP treatment resulted in substantial inflammatory infiltration into the alveoli and peri-bronchial regions. Moderate amounts of haemorrhage were also observed. Based on the histopathological data we chose to analyse mRNA up regulation in the lung tissue and systemic release of inflammatory mediators 24 hr after exposure to the ZnO NP solutions. Although we did not record increased eotaxin in the sera of mice treated with ZnO NPs, there was a significant up-regulation of eotaxin mRNA in mice treated with pristine 30 nm ZnO NPs indicating a potential eosinophilia. The discrepancies observed in the mRNA up regulation levels of the pro-inflammatory genes and the release of the same cytokines in the serum may be attributed to the fact that the Zno NPs may have stressed other tissue types besides the lung as previously reported by Gao and co-workers [35]. The lung environment may promote dissolution of ZnO NPs, which might result in lung injury [33]. ZnO NPs also caused a significant systemic release of pro-inflammatory serum chemokines including MCP-1, RANTES and IP-10, all associated with the recruitment of inflammatory cells to sites of inflammation. Cumulative inhaled ZnO NP doses of 10.9 mg/kg (306 μg/mouse) over 13 week exposure period was shown to elicit minimal pulmonary inflammation without causing significant release of pro-inflammatory markers in a recent animal study [49]. The preliminary results of our *in vivo* study highlight the inflammatory potential of inhaled ZnO NPs via intranasal installation, although the overall ZnO NP burden delivered is instantaneous and is relatively low with 5 mg/kg (84 μg/mouse). This highlights that the route and rate at which ZnO NPs are delivered *in vivo* may influence their toxic potential rather than the dose delivered. Similar observations have been previously suggested for poorly soluble titanium dioxide NPs [50]. In contrast to the *in vitro* studies no clear differences could be observed in the inflammatory profile of the two differentially dispersed 30 nm ZnO NP solutions. This may be explained by the complexity of the lung environment in terms of its cellular and protein diversity which may compound the bio-reactivity of NPs. Future detailed studies analysing the long term exposure of instilled NPs via the intranasal route are in progress.

## Conclusions

In summary, we have shown that the presence of ZnO NPs with low solubility in the cellular micro-environment helps initiate a strong antioxidant response as evident by the gradual up-regulation of the HO-1 gene. This is complemented by increased IL-8 gene expression and subsequent release of this pro-inflammatory cytokine. These immunological changes highlight the reactive nature of ZnO NPs even when no immediate cytotoxicity is observed. Dispersal state and particle size influenced the overall cytotoxicity and cellular responses of these NPs. NAC pre-treatment of A549 cells confirmed a role for ROS generation as a result of the elevated intracellular Zn^2+^ and subsequent phosphorylation of p65 and p38 transcription factors. Consequently, ZnO NP mediated cytotoxicity may be the outcome of failure of cellular redox machinery to contain excessive ROS formation. Furthermore, we have for the first time reported the potential of ZnO NPs to cause pulmonary immunomodulation when exposed via intranasal instillation. Our study provides a better understanding of the pro-inflammatory effects of interaction of ZnO NPs with the cellular systems of the airways.

## Methods

### Nanoparticles

Industrially manufactured ZnO NPs and particulates (30, 80 and 200 nm) were supplied by Micronisers Pty Ltd (Melbourne, Australia). The 30 nm ZnO NPs corresponded to the OECD standard reference material NM-112. Two different solutions of ZnO NPs were used, namely pristine material and particles dispersed with 5% by weight of sodium polyacrylate (Orotan 731 DP) surfactant dispersant (designated as “ZnO” or “sZnO”, respectively). All particulate stock suspensions were prepared in de-ionised water (MilliQ systems, Millipore). Physical characterisation data estimating primary particle size and particulate size before and after agglomeration in cell culture medium, along with cryo-transmission electron microscopy images, have been previously published [22]. Solubility of ZnO NPs in cell culture medium with or without protein (10% fetal bovine serum) was assessed using Varian Liberty Series II Inductively Coupled Plasma Atomic Emission Spectrometer (Melbourne, Australia); as previously described [6].

### Cell culture and nanoparticle exposure

Human alveolar lung epithelial A549 cells obtained from the American Type Culture Collection (ATCC, USA) were cultured in RPMI-1640 medium (Sigma-Aldrich, USA) supplemented with 10% fetal bovine serum, 1% penicillin–streptomycin and L-glutamine. For the study of cytotoxic effects, 10,000 cells in 100 μL were seeded into 96-well tissue culture plates (Sarstedt, Germany). After allowing for overnight attachment, the cells were exposed to ZnO NP concentrations (0.2 – 200 μg/mL) for 24 hr. For the study of mRNA up-regulation, release of cytokines and immunoblotting experiments, 10^6^ cells per well seeded into 6-well tissue culture plates were used. Subsequently cells were exposed to a dose of 20 μg/mL of the ZnO NPs for 1, 2, 4, 6 or 24 hr. ZnCl_2_ was used as a non-engineered form of zinc particulate control.

### Cytotoxicity assays

Viability of A549 cells was assessed via MTS (Promega MTS CellTiter 96® aqueous kit, Promega, USA), and lactate dehydrogenase release (LDH) (CytoTox 96® Non-Radioactive Cytotoxicity Assay Promega, USA) assays. At the end of the 24 hr exposure period, 50 μL of the supernatant was transferred to another 96-well flat-bottom plate. The NP-containing media in the plates with cells was then replaced with fresh medium containing MTS reagent and the plates were further incubated at 37°C for 3 hr and read at 490 nm (VersaMax, Molecular Devices, USA). ZnO particulates with MTS reagent alone provided readings of cell-free blank controls. The 50 μL supernatant sample was then mixed with an equal amount of LDH substrate mix. Following incubation at room temperature for 30 min, the absorbance was recorded at 490 nm. Supernatant from wells exposed to 5 μL lysis solution were used as the 100% LDH release positive control. Wells containing cells exposed to medium-only served as spontaneous LDH release control. Data were obtained from five independent experiments, each performed in triplicate.

### RNA isolation and quantitative-PCR (q-PCR)

Total RNA isolation from treated A549 cells was carried out using the ISOLATE RNA Mini Kit (Bioline, USA) as per manufacturer’s instructions. Concentrations of extracted RNA samples were determined using a Nanodrop 8000 spectrophotometer (Thermo Scientific, Germany) and reverse transcribed into cDNA using the Tetro cDNA Synthesis kit (Bioline, USA). Changes in expression levels of the pro-inflammatory cytokine IL-8 and the oxidative stress response enzyme heme oxygenase-1 (HO-1) were determined using q-PCR using the SsoAdvanced ™SYBR ® Green Supermix (BioRad, USA) reaction mixture containing specific primers. Primers used were: HO-1 primers, sense 5′-CGCCTTCCTGCTCAACATT-3′ and antisense 5′-TGTGTTCCTCTGTCAGCATCAC-3′. IL-8: sense 5′-GGCACAAACTTTCAGAGACAG-3′ and antisense 5′-ACACAGAGCTGCAGAAATCAGG-3′. The housekeeping gene used was ubiquitin, sense 5′-GCAAGCTACAATAATGGGGC-3′ and antisense 5′- TGTAAATGCAACCTTAGGTGGT-3′. q-PCR was performed using the Piko Real real-time PCR System (Thermo Scientific, Germany). Thermal cycle parameters used were 7 min at 95°C, followed by 45 cycles (95°C, 15 sec; 55°C, 15 sec; 72°C, 30 sec). Data were analysed using relative quantitation.

### IL-8 release

ZnO NPs induced IL-8 release was assessed using enzyme-linked immunosorbant assay (ELISA) in cell supernatants of A549 cells exposed to ZnO NPs using the BD OptEIA human IL-8 ELISA kit (BD Systems, USA) as per manufacturer’s instructions. Three independent experiments were performed, each with triplicate samples.

### Effect of sulfhydryl antioxidant

A549 cells were pre-treated with 5 mM of the sulfhydryl antioxidant N-acetylcysteine (NAC) (Sigma-Aldrich, USA) for 1 hr before exposure to ZnO NPs. The NAC dose was confirmed to be non-cytotoxic to A549 cells using the MTS assay.

### Cell signalling: immunoblotting for NFκB, p-38 MAPK pathway activation

At the end of the *in vitro* NP exposures for 1, 6 or 24 hr, the cells were washed with PBS and lysed using NP40 cell lysis buffer (Invitrogen, USA) supplemented with 1 mM phenylmethylsulfonyl fluoride (PMSF) and protease inhibitor cocktail (Sigma-Aldrich, USA). The cell lysates obtained were the subjected to SDS-PAGE followed by transfer onto a nitrocellulose membrane using the semi-dry transfer system (BioRad, USA). After blocking with 1% Superblock (Sigma-Aldrich, USA) blocking solution for 1 hr, the membranes were probed overnight with specific primary antibodies: Phospho p38(Thr180/Tyr182), p38 MAPK antibody, along with phospho-NFκB p65(Ser536) or NFκB p65 antibodies (Cell Signalling, USA), followed by washing with PBS. Proteins were detected using horseradish peroxidase-tagged secondary antibodies (Cell Signalling, USA) and enhanced chemiluminescence substrate (Pierce, USA), followed by exposure to photographic film (GE Healthcare, Australia).

### Intranasal instillation of ZnO NPs in mice

Female Balb/c mice (6–8 weeks old) purchased from the Animal Resource Centre (WA, Australia) were randomly assigned to four groups (n = 12) based on body weight (14–19 gm). 30 nm pristine and surfactant-dispersed ZnO NP solutions were prepared in sterile water. The dose used for the study was 5 mg/kg (body weight). 50 μL of the dose was delivered into each nostril of the mice held in supine position, using a 200 μL pipette tip. Two out of the four experimental groups served as controls including groups treated with a non-lethal dose of 0.5 mg/kg lipopolysaccharide (LPS) or water (vehicle control). At the end of 24 hr exposure period mice were killed and peripheral blood and lungs harvested from each animal.

Serum prepared from the collected blood samples was pooled for animals from each group and used for the mouse proteome array (R&D Biosystems) as per manufacturer’s instructions. Small sections of the tissue obtained were also used for RNA extraction and quantification of mRNA levels of pro-inflammatory markers including: eotaxin, sense 5′-AGAGGCTGAGATCCAAGCAG-3′ and antisense 5′-CAGATCTCTTTGCCCAACCT-3′, TNFα, sense 5′- TACTGAACTTCGGGGTGATTGGTCC-3′ and antisense 5′-CAGCCTTGTCCCTTGAAGAGAACC-3′, MCP-1, sense 5′-ACCACAGTCCATGCCATCAC-3′ and antisense 5′-TTGAGGTGGTTGTGGAAAAG-3′ and the housekeeping gene used was β actin, sense 5′-CGAGCGTGGCTACAGCTTCA-3′ and antisense 5′-AGGAAGAGGATGCGGCAGTG-3′. The remaining intact lung tissue was used for histological analysis. H&E staining was performed on formalin-fixed lung sections embedded in paraffin and sliced into 5 μm thick sections and examined under a light microscope. Animal experiments were approved by the animal ethics committee at James Cook University.

### Statistics

Data are presented as mean ± standard error of mean (SEM) and was analysed using two way ANOVA for cytotoxicity assays or one-way ANOVA for all other analyses, followed by Bonferroni post-hoc test (Prism 6.0, GraphPad Software, USA), with a p value <0.05 considered as significant.

## Additional file

Additional file 1: Table S1. Dissolution rates of ZnO NPs used in the present study. ICP-AES was performed on dialysates of ZnO NPs 200 μg/mL incubated in RPMI-1640 medium without protein for 24 hr. *Data are adapted from (Feltis et al., 2012) and represent values obtained from identical experiments carried out in the presence of 10% fetal bovine serum supplemented RPMI-1640 medium.

## Abbreviations

NP: Nanoparticles
ZnO NP: Zinc oxide nanoparticles
ROS: Reactive oxygen species
LDH: Lactate dehydrogenase
HO-1: Hemeoxygenase-1
IL-8: Interleukin 8
MCP-1: Monocyte chemo attractant protein 1
TNFα: Tumour necrosis factor alpha
RANTES: Regulated on activation, normal T cell expressed and secreted chemokin
NAC: N-acetylcysteine
PMSF: Phenylmethylsulfonyl fluoride
