# Supplementary material for: Investigating the immunomodulatory nature of zinc oxide nanoparticles at sub-cytotoxic levels in vitro and after intranasal instillation in vivo
Source: J Nanobiotechnology. 2015 Feb 3;13:6. doi: 10.1186/s12951-015-0067-7 (PMC4324663; doi:10.1186/s12951-015-0067-7)
Supplement: Additional file 1: Table S1. — Dissolution rates of ZnO NPs used in the present study. ICP-AES was performed on dialysates of ZnO NPs 200 μg/mL incubated in RPMI-1640 medium without protein for 24 hr. *Data are adapted from (Feltis et al., 2012) and represent values obtained from identical experiments carried out in the presence of 10% fetal bovine serum supplemented RPMI-1640 medium. [file 12951_2015_67_MOESM1_ESM.pdf]

Supplementary

Table 1: Dissolution rates of ZnO NPs used in the present study. ICP-AES was performed on dialysates of ZnO NPs 200 µg/mL incubated in RPMI-1640 medium without protein for 24hr. *Data are adapted from (Feltis et al., 2012) and represent values obtained from identical experiments carried out in the presence of 10% fetal bovine serum supplemented RPMI-1640 medium

Zinc oxide nanoparticle	Zinc concentration (µg.mL^{-1})	
	RPMI-1640 medium without protein	RPMI-1640 medium supplemented with10% fetal bovine serum
ZnO-NP 30	1.2	1.85*
ZnO-NP s30	1.8	1.44*
ZnO-NP 80	1.3	NA
ZnO-NP s80	2.2	NA
ZnO-NP 200	1	2.8*
ZnO-NP s200	0.8	2.32*
Basal medium	0.04	NA
